# Cerebral Blood Transit in Sickle Cell Anemia

**DOI:** 10.1002/jmri.70382

**Published:** 2026-05-28

**Authors:** Wesley T. Richerson, Megan A. Aumann, Alexander K. Song, Samantha Davis, Lauren Milner, Maria Garza, Dann Martin, L. Taylor Davis, Sumit Pruthi, Lori C. Jordan, Manus J. Donahue

**Affiliations:** ^1^ Department of Neurology Vanderbilt University Medical Center Nashville Tennessee USA; ^2^ Division of Pediatric Neurology, Department of Pediatrics Vanderbilt University Medical Center Nashville Tennessee USA; ^3^ Department of Radiology and Radiological Sciences Vanderbilt University Medical Center Nashville Tennessee USA; ^4^ Department of Psychiatry and Behavioral Sciences Vanderbilt University Medical Center Nashville Tennessee USA

**Keywords:** arterial spin labeling, arterial transit time, bolus arrival time, capillary shunting, cerebral hemodynamics, sickle cell anemia

## Abstract

**Background:**

Sickle cell anemia (SCA) patients upregulate cerebral blood flow to compensate for decreased arterial oxygen content. Such hyperemic conditions can manifest as venous hyperintense signal on arterial spin labeling (ASL) MRI, which may reflect faster capillary blood transit, altered oxygen extraction fraction (OEF), and infarct risk.

**Purpose:**

To implement multi‐delay ASL to quantify cerebral arterial arrival time (AAT), arterial transit time (ATT) to tissue, and venous arrival time (VAT) and to test whether reductions in these parameters are present in SCA and relate to condition severity.

**Study Type:**

Prospective, cross‐sectional.

**Subjects:**

Adults with SCA (*n* = 40; age = 28.1 ± 7.1 years; sex = 47.5% female) and nonanemic controls (*n* = 24; age = 30.6 ± 6.6 years; sex = 37.5% male).

**Field Strength/Sequence:**

3 T 3D *T*_1_‐weighted, 3D *T*_2_‐FLuid‐Attenuated‐Inversion‐Recovery, 2D *T*_2_‐relaxation‐under‐spin‐tagging (TRUST), 3D pseudo‐Continuous ASL (pCASL) Gradient‐And‐Spin‐Echo, and 2D multi‐delay (delay range = 200–3200 ms) Pulsed ASL (PASL).

**Assessment:**

Multi‐delay PASL was used to quantify AAT, gray matter ATT, VAT, and labeled venous blood volume. Venous hyperintensities and OEF were quantified from 3D pCASL and TRUST, respectively. Infarcts and vasculopathy were assessed from radiological review.

**Statistical Tests:**

AAT, gray matter ATT, VAT, and labeled venous blood volume were evaluated using a Wilcoxon rank‐sum test; ANOVA assessed relationships with venous hyperintense signal. Continuous variable relationships were assessed using Spearman rank‐correlation (significance: two‐sided *p* < 0.05).

**Results:**

Venous labeled blood volume was significantly increased in SCA (median = 0.015 mL; IQR = 0.0087–0.023 mL) versus control (median = 0.0047 mL; IQR = 0.0032–0.0092 mL) participants, whereas VAT was significantly reduced in SCA (median = 2.53 s; IQR = 2.23–2.72 s) versus control (median = 2.75 s; IQR = 2.63–2.80 s) participants. AAT, gray matter ATT, and VAT values were not significantly related to OEF (AAT: *p* = 0.38; gray matter ATT: *p* = 0.24; VAT: *p* = 0.48) or prior infarct (AAT: *p* = 0.99; gray matter ATT: *p* = 0.88; VAT: *p* = 0.98). AAT and gray matter ATT, but not VAT (*p* = 0.153), were directly related to hemoglobin.

**Conclusion:**

SCA patients had elevated labeled venous blood volume and shorter VAT. Differential relationships between blood transit and hemoglobin across vascular compartments provides support for venous blood kinetics not representing a simple sequela of anemia severity.

**Evidence Level:**

2.

**Technical Stage of Development:**

2.

## Plain Language Summary

Sickle cell anemia is an inherited blood disorder characterized by reduced levels of the oxygen carrying molecule, hemoglobin, which can place patients at increased risk for stroke. This work uses new magnetic resonance imaging (MRI) approaches to show that to compensate for such anemia, the blood flow to the brain is higher, and importantly, the time it takes for blood to move through the brain vasculature is shorter. These findings provide further motivation for evaluating blood transit as a marker of brain health and stroke risk, and possibly, as an outcome measure in emerging trials of hemoglobin augmentation therapies.

## 1 Introduction

Sickle cell anemia (SCA) is the most common genetic blood disorder, caused by a point mutation in the gene encoding the hemoglobin *β* subunit and resulting in red cell hemolysis, anemia, and decreased arterial oxygen content (CaO_2_; mL O_2_/100 mL blood) [1, 2]. SCA lifespan remains 40–50 years even for patients in high resource settings with access to hemoglobin augmentation therapies [2], however, the SCA treatment landscape is promising, with newly‐approved gene therapies and stem cell transplants offering curative alternatives to more conservative oral hydroxyurea and monthly blood transfusions [3]. However, such curative therapies are costly and carry risks related to nonengraftment, malignancy, and death [3]. The fundamental question in this population is whether the benefits of achieving cure outweigh the procedural risks. To address this question, a more precise understanding of the pathophysiology of SCA, as well as personalized assessments that can be used to triage patients for appropriate disease‐modifying therapies, are needed [4].

Patients with SCA are at increased risk of stroke and cognitive impairment, and stroke risk can help to triage patients for more aggressive therapies [5]. While elevated middle cerebral artery (MCA) velocity has been demonstrated to portend stroke in children, no clinical exam for conferring SCI development risk exists for adults. Importantly, despite the documented 50% incidence of overt or silent infarct by age 30 years, fewer than 15% of patients have conventional ischemic risk factors such as intracranial or extracranial large artery stenosis or hypercholesteremia, indicating that other factors contribute [6, 7, 8].

Recent work on parenchymal compensation has helped to inform possible candidate risk factors [9, 10, 11, 12]. Specifically, to maintain cerebral oxygen delivery, cerebral blood flow (CBF; mL blood/100 g tissue/min) is upregulated to compensate for reduced CaO_2_ [9, 10, 13]. Yet, even patients with extreme hyperemia and similar levels of anemia develop recurrent infarcts, suggesting that infarcts are not a simple sequela of limited cerebral oxygen delivery in the setting of anemia. Indeed, under similar cerebral perfusion pressures, increased CBF generally co‐localizes with increased blood flow velocity [9, 14]. Increased or heterogeneous capillary flow velocities may limit red cell residency times within the capillary bed, decreasing time for oxygen offloading [15]. These effects have been modeled using exogenous contrast agents and MRI, whereby it has been shown that capillary transit time heterogeneity increases across a range of conditions, including neurodegeneration and cerebrovascular disease [16, 17].

While perfusion‐weighted brain MRIs with exogenous contrast agents are not frequently performed in patients with SCA, dural sinus hyperintensities on noninvasive perfusion‐weighted arterial spin labeling (ASL) MRI have been observed from multiple groups in patients with SCA, which are consistent with abnormal water exchange between the capillaries and tissue or accelerated blood transit [9, 18, 19, 20, 21, 22]. These signal hyperintensities have been shown to be overrepresented in the setting of anemia [23, 24], correlate directly with independent measures of large‐vessel flow velocities [24], relate to infarct history [18], and reduce following transfusion or stem cell transplant‐induced hemoglobin augmentation therapies [19, 25]. This work collectively supports the possibility that these sinus hyperintensities reflect higher flow velocities, relate to indicators of condition severity, and can be modified by hemoglobin‐augmentation therapies.

However, most prior work has leveraged single post‐labeling delay ASL data, which provided a promising, albeit coarse and ordinal shunting score of blood transit [18, 24]. This work utilizes a time‐efficient multi‐delay pulsed ASL method, designed to assess early arterial and later venous compartments, in a prospective, cross‐sectional study of vascular transit times in adults with and without SCA. The aim of this study is to test the hypotheses that venous arrival times (VATs) are shorter, and the volume of labeled blood greater, in SCA patients relative to controls, and that the labeled venous blood volume and VATs correlate with ordinal scoring of venous shunting used in prior studies [18, 19, 26]. A secondary goal is to evaluate whether reduced transit times and increased labeled venous blood volumes associate with clinical indicators of condition severity, including total hemoglobin and infarct history. The ultimate goal of this work is to develop a sensitive biomarker of cerebral infarct risk in SCA patients.

## 2 Materials and Methods

### 2.1 Participants

Protocols were approved by the Vanderbilt Health Institutional Review Board (#220151), and participants provided informed consent for this prospective, cross‐sectional study. Inclusion criteria: SCA patients (hemoglobin‐SS (Hb‐SS) or hemoglobin‐Sβ0‐thalassemia (Hb‐Sβ0) phenotype) and nonanemic (total hemoglobin > 12 g/dL) hemoglobin‐AA (HbAA) controls, age range = 18–50 years. Common exclusion criteria were 3‐Tesla prohibited implant and history of any major independent neurological, vascular, or psychiatric conditions. SCA patients having received stem cell therapy were excluded; however, patients were not excluded based on prior infarcts, vasculopathy or difference in noncurative treatments (hydroxyurea or transfusion), as these variables were evaluated for potential relevance to imaging assessments. Control participants with asymptomatic; nonspecific white matter lesions were also not excluded, as these are not uncommon with increasing age and the cohort was deemed to be representative [27].

### 2.2 Hematological Measures

Hemoglobin type was evaluated by venipuncture and electrophoresis. Total hemoglobin level, as well as hemoglobin‐S percentage and arterial oxygen saturation, were measured in participants within 7 days of the MRI scan. In the subgroup of SCA participants on blood transfusion, the hemoglobin and imaging measurements were made more than 3 weeks since prior transfusion when hemoglobin was near nadir. Hemoglobin and arterial oxygen saturation were used to calculate CaO_2_ using a binding coefficient of 1.34 mL O_2_/g Hb.

### 2.3 MRI and Angiography (MRA)

MRI data were acquired at 3 T (Philips Healthcare, Best, the Netherlands) with 32‐channel phased‐array head coil reception. For both cohorts, a noninvasive head MRI and MRA protocol was applied including: 3D *T*_1_‐weighted (magnetization‐prepared‐rapid‐gradient‐echo; spatial resolution = 1 × 1 × 1 mm; 3D turbo‐fast‐echo; compressed sensing; repetition time (TR)/echo time (TE) = 6.7/3.1 ms), 3D *T*_2_‐weighted FLuid Attenuated Inversion Recovery (FLAIR) (spatial resolution = 1.1 × 1.1 × 1.1 mm; turbo‐inversion‐recovery; compressed sensing; TR/inversion time (TI)/TE = 4800/1650/340 ms), and intracranial time‐of‐flight (TOF) MRA (spatial resolution = 0.5 × 0.8 × 1.4 mm; gradient echo; TR/TE = 23/3.5 ms).

**FIGURE 1:**
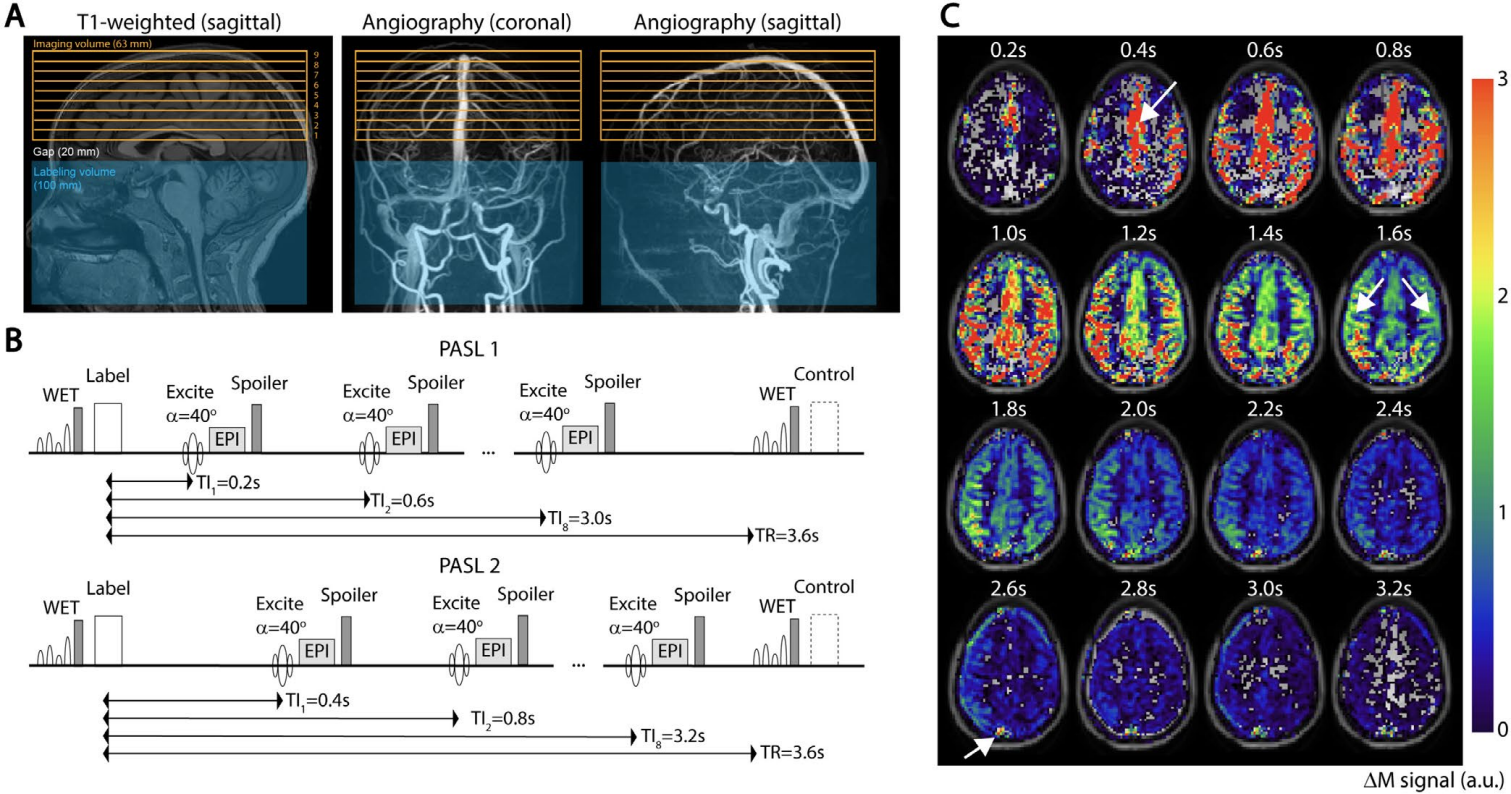
MRI acquisition and example single participant data. (A) The imaging volume is planned with the most distal slice above the most distal aspect of the superior sagittal sinus, with coverage extending proximally over the majority of the bi‐frontal and bi‐parietal lobes, where infarcts are most common in patients with sickle cell anemia. Imaging and labeling volumes are overlaid on *T*_1_‐weighted (left) MRI and coronal and sagittal maximum intensity angiography projections (right). (B) Given the high temporal resolution and temporal range required, a pulsed arterial spin labeling (PASL) module is utilized with an intermediate flip angle = 40° Look‐Locker readout, with eight inversion times staggered by 0.2 s acquired in two sequential acquisitions, ultimately extending from 0.2 to 3.2 s. (C) Representative multi‐delay data from a single slice of one participant highlights sensitivity to earlier arterial flow (e.g., 0.4 s; white arrow), gray matter perfusion (e.g., 1.6 s; white arrows), and late venous arrival in the dural sinus (e.g., 2.6 s; white arrow). Note regional asymmetry in arrival times and perfusion, which are common in sickle cell anemia and are assessed in this study.

To quantify CBF, arterial arrival time (AAT; i.e., time for labeled arterial water to arrive in the pre‐capillary compartment of the imaging voxel), gray matter arterial transit time (ATT; i.e., time for labeled blood water to exchange with gray matter tissue water), and venous arrival time (VAT; i.e., time for labeled blood water to arrive in veins), and labeled venous blood volume, a 2D echo‐planar‐imaging (EPI) multi‐delay pulsed ASL (PASL) sequence was utilized (spatial resolution = 3 × 3 × 7 mm; Look‐Locker readout [flip angle = 40°]; signal targeting by alternating radiofrequency [STAR] pulsed labeling; repetitions = 25; total acquisition time = 6 min 56 s) (Figure 1). The readout acquired 16 inversion times (TIs) equally spaced from 200 to 3200 ms, collected in two interleaved 3 min 28 s acquisitions. The first Look‐Locker acquisition collected eight TIs equally spaced between 200 and 3000 ms, while the second acquisition collected eight TIs equally spaced between 400 and 3200 ms. Identical calibration, shimming, and gain settings were used for both scans. PASL was used owing to its known increased sensitivity to arrival time, given the short labeling duration. 2D EPI, rather than 3D GRadient‐And‐Spin‐Echo (GRASE), was chosen given signal smoothing that occurs in the phase‐encode direction of 3D GRASE for nonheavily segmented readouts and the ability of EPI to be readily incorporated in a Look‐Locker readout [28, 29]. An *M*_0_ image was collected with the same geometry parameters but spin labeling removed and TR = 15 s.

To enable ordinal scoring of venous signal hyperintensity analogous to prior studies [18, 20, 24, 30] for additional comparison, a single‐delay pseudo‐continuous ASL (pCASL) sequence was applied (labeling duration = 1800 ms, post‐labeling delay = 2000 ms, spatial resolution = 3 x 3 x 7 mm, 20 slices, 3D GRASE readout, scan duration = 4 min 54 s, repeats = 5, five‐fold readout segmentation with turbo‐spin‐echo factor = 19 and EPI factor = 15). An M_0_ image was collected with the same geometry parameters, but labeling was removed and TR = 15 s.

To measure whole‐brain OEF, *T*_2_‐relaxation‐under‐spin‐tagging (TRUST) was applied (spatial resolution = 3.4 × 3.4 × 5 mm, τ‐CPMG = 10 ms, effective echo times (eTE) = 0, 40, 80, 160 ms, TR/TE = 1978/3.6 ms, averages per eTE = 6) using a previously‐reported protocol evaluated for reproducibility [31, 32]. Control and venous blood‐labeled TRUST slices were planned with the slice transecting the superior sagittal sinus approximately 20 mm distal to the torcula.

### 2.4 Anatomical Imaging and Angiography Analysis

*T*_*1*_‐weighted, *T*_*2*_‐weighted FLAIR, and MRA were evaluated by three neuroradiologists (D.M.: experience = 6 years; S.P.: experience = 16 years; L.T.D: experience = 12 years). MRA was used to assess and grade intracranial vasculopathy and was assessed as 0%–50% (no stenosis), 51%–69% (mild stenosis), 70%–99% (significant stenosis), or occlusion. Intracranial segments of the ICAs, basilar artery, and first segments of the anterior, middle, and posterior cerebral arteries were assessed. *T*_*1*_‐weighted and FLAIR were used to assess overt infarcts and SCI occurrence. Infarcts were judged to be silent upon review by a neurologist (L.J.; experience = 21 years). *T*_*1*_‐weighted images were used for registration between PASL and an MNI152 *T*_*1*_‐weighted 2 mm atlas and for tissue segmentation using advanced normalization tools (ANTs) [33] and the FMRIB Software Library (FSL) [34, 35, 36] (Supporting Information: Methods).

### 2.5 Transit Time Analysis

PASL data were pair‐wise subtracted and motion‐corrected to obtain the ΔM image across each repetition and inversion time, after which each ΔM image was normalized by the *M*_0_ equilibrium magnetization image to calculate the CBF‐weighted image (ΔM/M_0_). Each CBF‐weighted image was averaged across the 25 repeated measurements for each participant at each inversion time. Blood water *T*_1_ was calculated in the SCA group using the hematocrit recorded from electrophoresis with the calibration curve from Lu et al. [37], as unlike for venous blood water [38], there is currently no separate *T*_1_ calibration model for SCA arterial blood water and, in contrast to *T*_2_, hemoglobin type is believed to have only a small effect on arterial blood water *T*_1_ [39].

All fitting was performed in the native subject space. First, to calculate the CBF, arterial arrival time (AAT), and gray matter ATT, the mean CBF‐weighted time series was supplied to FSL‐BASIL [40], where the CBF‐weighted signal was modeled with the flow‐modified Bloch equation with intravascular arterial and gray matter tissue contributions. The tissue contribution was modeled as,

(1) ∆M/M0=0TI<ATT2αfe−TI·R1,bloodR1app,eff−R1,blood1−e−TI−ATTR1app,eff−R1,blood−1ATT≤TI≤ATT+τt2αfe−ATT+τt·R1,bloodR1app,eff−R1,blood1−e−TI−ATTR1app,eff−R1,blood−1e−TI−ATT−τtR1app,effATT+τt<TI

where α is the labeling efficiency (set to 0.98 for PASL [29]), *τ_t_* is the bolus length (set to 0.7 s for tissue; see Discussion), *TI* is the inversion time (s), *R*_1,blood_ = 1/*T*_1,blood_ is the calculated, hematocrit‐corrected arterial blood longitudinal relaxation rate (s^−1^), *ATT* is the arterial transit time (s), and *f* is the perfusion (mL/g/s). Note that a factor of 6000 is required to convert *f* to *CBF* in conventional units of mL/100 g/min. The apparent Look‐Locker relaxation rate of tissue, as proposed by Günther et al. [28], is,

(2) $$R_{1\mathrm{app},\mathrm{eff}}=R_{1,t}+\frac{f}{\lambda }-\frac{\ln\left(\cos\left(\mathrm{FA}\right)\right)}{\mathrm{\Delta TI}}$$

where *R*_1,t_ is the 3 Tesla relaxation rate of tissue (set to 0.833 s^−1^) [37], *λ* is the tissue partition coefficient (set to 0.98 mL/g) [41], *FA* is the flip angle of the Look‐Locker readout (40° = 2π/9 rad), and Δ*TI* is the time between inversion pulses in each PASL acquisition (prescribed as 0.4 s). Equations (1) and (2) were used to quantify the voxel‐wise CBF and ATT across the brain with standard FSL‐BASIL variational Bayesian fitting, additionally applying partial volume correction within the pipeline [28, 40, 42, 43] (Supporting Information). Additionally, within FSL‐BASIL [40], the arterial intravascular signal was modeled during this process within each of these voxels to estimate AAT using:

(3) $$∆M/M_{0}=\left\{\begin{array}{cc} 0 & \mathrm{TI}<\mathrm{AAT}\\ 2\alpha \left({\mathrm{CBV}}_{a}\right)e^{-\mathrm{TI}\cdot R_{1,\text{blood}}} & \mathrm{AAT}\leq \mathrm{TI}\leq \mathrm{AAT}+\tau_{a}\;\\ 0 & \mathrm{AAT}+\tau_{a}<\mathrm{TI} \end{array}\right.$$

where *AAT* is the arterial arrival time (i.e., time for labeled blood water to arrive in the voxel), *CBV*_*a*_ is the arterial blood volume, and *τ*_a_ is the bolus length (set to 0.7 s; see Discussion).

Since each parameter in FSL‐BASIL is estimated using variational Bayesian fitting [40], each parameter is estimated as a posterior normal distribution with a mean and standard deviation. Voxels with a mean CBV_a_ three standard deviations above zero were identified as voxels with predominate arterial contributions and used as the arterial mask. AAT was then averaged within this mask to obtain the subject AAT.

Next, the intravascular VAT values were calculated separately using a similar intravascular model applied to the superior sagittal sinus:

(4) $$∆M/M_{0}=\left\{\begin{array}{cc} 0 & \mathrm{TI}<\mathrm{VAT}\\ 2\alpha \left({\mathrm{CBV}}_{v}\right)e^{-\mathrm{TI}\cdot R_{1,\text{blood}}} & \mathrm{VAT}\leq \mathrm{TI}\leq \mathrm{VAT}+\tau_{v}\\ 0 & \mathrm{VAT}+\tau_{v}<\mathrm{TI} \end{array}\right.$$

here *VAT* is the venous arrival time, *CBV*_*v*_ is the venous labeled blood volume, and *τ_v_* is the bolus length (a parameter which was fit for). The equation was applied to a superior sagittal sinus mask, and the identification of this region is outlined in detail in the Supporting Information. Labeled venous blood volume (mL) was calculated by multiplying the voxel size in mL by the estimated labeled blood water percentage. Values preserved for hypothesis testing were the AAT, ATT, VAT, and labeled venous blood volume.

### 2.6 CBF and Ordinal Venous Hyperintensity Score Analysis

To enable comparison with ordinal methods used in prior work from different groups, pCASL CBF was quantified from the single‐delay pCASL 3D GRASE data with identical methods described in the literature [9] and ordinal venous hyperintensity scoring (score = 0, 1, or 2) was performed using previously described criteria [24] (Supporting Information).

### 2.7 OEF Analysis

Using the TRUST data and following previously reported procedures [31, 44], the sagittal sinus blood water CPMG transverse relaxation time (*T*_*2*_) was calculated from the pairwise subtracted control and venous labeled slices and a single exponential decay model fit to this difference signal as a function of eTE. For completeness, venous oxygenation (Y_v_) was calculated using four separate models (full Bovine [31], abbreviated Hb‐AA [45], full Hb‐F [46] and abbreviated Hb‐S [47]), as each have been published and have unique considerations in the setting of anemia and hemoglobinopathies. Supratentorial OEF for each model was then calculated as the fractional difference between arterial oxygenation measured from pulse oximetry and venous oxygenation calculated here from TRUST.

### 2.8 Statistical Analysis

First, continuous demographic and categorical variables were compared between groups using a Wilcoxon rank‐sum test and chi‐squared test, respectively. Groups were required to be matched for basic demographic variables such as age and sex with two‐sided *p* > 0.05. Demographic, hematologic, and clinical variables for each group are presented as median and inter‐quartile range (IQR) for continuous variables and frequency for categorical or ordinal variables, unless otherwise noted.

To test the overarching hypothesis that vascular transit variables were reduced in SCA versus control participants, a Wilcoxon rank‐sum test was performed to compare values for each cohort, separately for arterial (AAT), gray matter tissue (ATT), and venous (VAT) transit time measures, as well as separately for the labeled venous blood volume metric. To understand whether the quantitative vascular transit measures from PASL related to venous hyperintensity scoring from single‐delay pCASL (values reported in the literature), each participant was grouped by hyperintensity score quantified from pCASL (i.e., 0, 1, or 2) and a one‐way ANOVA was applied for the vascular transit values from PASL between each group; F‐statistics and two‐sided *p*‐values are reported. Finally, relationships with previously proposed indicators of condition severity such as OEF, prior SCI, and total hemoglobin were evaluated. The relationships between the HbF model OEF and venous labeled blood volume, AAT, gray matter ATT and VAT were evaluated in the SCA cohort using a Spearman correlation test, with the same test repeated for all available OEF models for completeness (Supporting Information). The relationship between prior infarct occurrence (stroke or SCI) and venous labeled blood volume, AAT, gray matter ATT and VAT were assessed using a Wilcoxon rank‐sum test. The relationship between AAT, gray matter ATT and VAT with hemoglobin was analyzed in the SCA cohort using a Spearman correlation test, separately for each vascular compartment. For all analyses, significance criterion was defined as two‐sided *p* < 0.05.

## 3 Results

### 3.1 Participants

40 SCA (age = 28.1 ± 7.1 years; sex = 47.5% male) and 24 control (age = 30.6 ± 6.6 years; sex = 37.5% male) participants provided informed consent and met all inclusion criteria. Age (*p* = 0.125) and sex (*p* = 0.440) were not significantly different between groups. 60.0% of SCA and 12.5% of control participants had SCIs. 17.5% of SCA participants had a prior stroke; 12.5% had significant intracranial stenosis with moyamoya vasculopathy. Of the seven participants with a prior stroke, four were also diagnosed with moyamoya vasculopathy. Additional details are summarized in Table 1 and sub‐analyses for moyamoya comorbidity included in Supporting Information.

**TABLE 1:** Demographic and hemodynamic characteristics.

	Sickle cell anemia (SCA)	Control	*p*
*Demographic characteristics*			
Number	40	24	
Sex (% male)	47.5	37.5	*p* = 0.440
Age (years)	26.5 (22.5–32.0)	29.5 (25.5–34.5)	*p* = 0.125
*Hematological*			
Hemoglobin (g/dL)	8.8 (8.0–10.0)	13.3 (12.1–13.9)*	*p* < 0.001
Hemoglobin S (%)	75.0 (59.7–82.9)	0.0	*p* < 0.001
CaO_2_ (mL O_2_/dL)	10.8 (10.1–12.9)	17.5 (15.8–17.8)*	*p* < 0.001
*Vascular disease*			
Moyamoya syndrome (%)	12.5	0	*p* < 0.001
Stroke (%)	17.5	0	*p* < 0.001
Silent cerebral infarct (%)	60.0	12.5	*p* < 0.001
1–5 silent cerebral infarcts (%)	32.5	12.5	
6–9 silent cerebral infarcts (%)	17.5	0	*p* < 0.001
10+ silent cerebral infarcts (%)	10.0	0	*p* < 0.001
Venous hyperintensity scoring (pCASL 3D‐GRASE)**			
Score = 0 (%)	22.5	50.0	*p* = 0.028
Score = 1 (%)	40.0	50.0	*p* = 0.270
Score = 2 (%)	35.0	0.0	*p* < 0.001
*Shunting and transit times (PASL 2D EPI)*			
Labeled venous blood volume (mL)	0.015 (0.0087–0.023)	0.0047 (0.0032–0.0092)	*p* < 0.001
Arterial arrival time (s)	0.35 (0.32–0.38)	0.37 (0.33–0.38)	*p* = 0.371
Gray matter arterial transit time (s)	0.58 (0.54–0.65)	0.64 (0.58–0.66)	*p* = 0.022
Venous arrival time (s)	2.53 (2.23–2.72)	2.75 (2.63–2.80)	*p* = 0.006
Oxygen extraction fraction***			
Full bovine model (%)	44.0 (41.1–48.3)	34.7 (32.6–38.7)	*p* < 0.001
Abbreviated HbA model (%)	40.2 (37.9–43.1)	38.0 (36.4–41.8)	*p* = 0.278
Full HbF model (%)	44.0 (42.0–47.4)	37.9 (36.3–41.8)	*p* < 0.001
Abbreviated HbS model (%)	29.7 (25.9–31.9)	38.0 (36.4–41.8)****	*p* < 0.001

*Note:* Data are presented as median (25th percentile–75th percentile). Uncorrected *p*‐values displayed. *Hemoglobin values collected in 18 controls. **pCASL venous hyperintensity score collected in 39 sickle cell participants. ***TRUST was collected in 18 age‐matched controls. ****Hemoglobin‐A model OEF used for control participants in comparison with Hemoglobin‐S model in SCA participants.

Abbreviations: 2D EPI, 2D echo‐planar imaging; 3D‐GRASE, 3D gradient‐and‐spin‐echo; PASL, pulse arterial spin labeling; pCASL, pseudo‐continuous arterial spin labeling.

### 3.2 Transit Times

**FIGURE 2:**
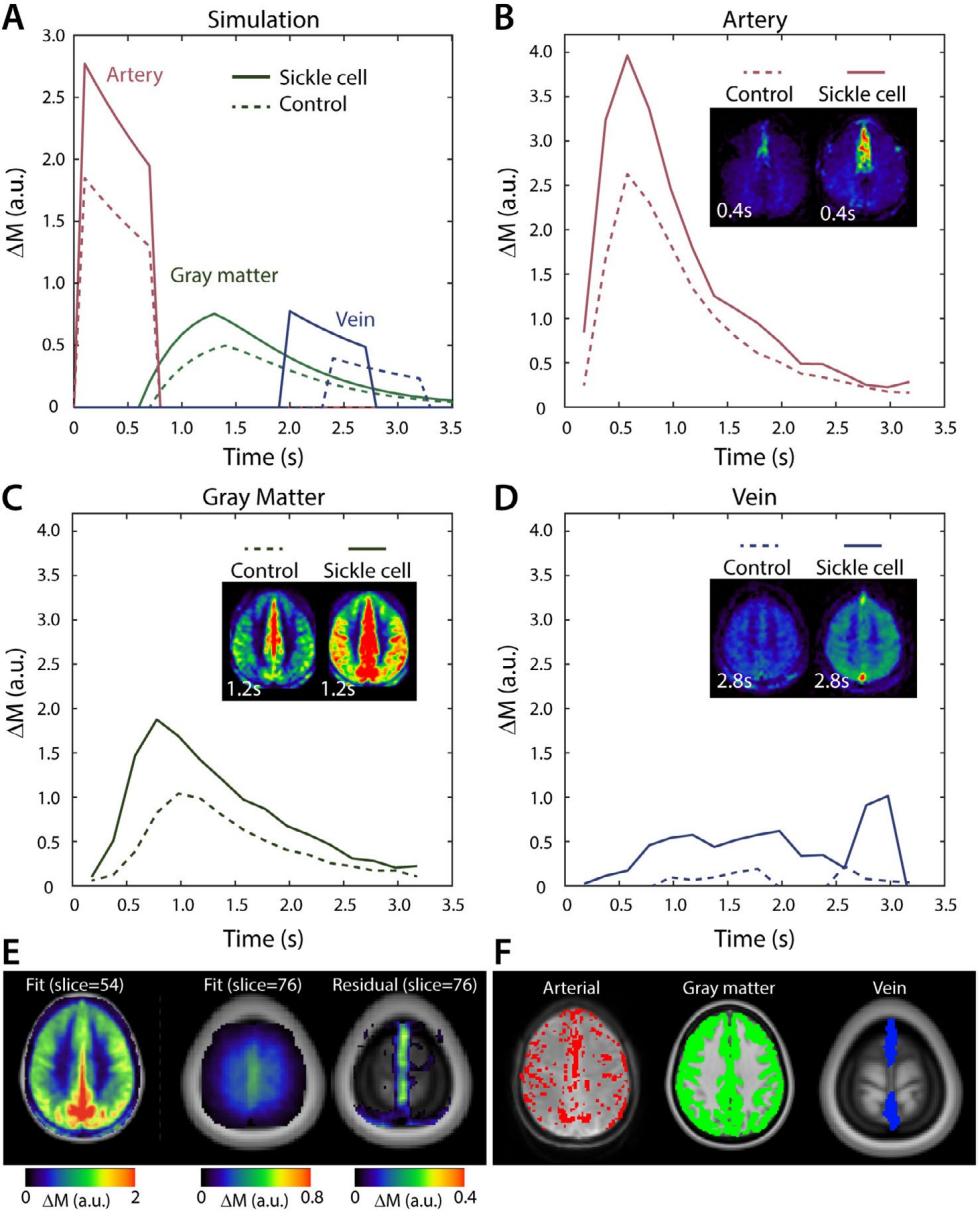
Kinetic modeling and representative arrival time data. (A) Simulations of kinetic curves from the multi‐compartment models, separately for hypothesized sickle cell and nonanemic control scenarios. Here, near‐immediate arrival of arterial blood differs minimally between cohorts, but longer transit differences in gray matter (0.6–0.8 s) and veins (2.0–2.4 s) are simulated, along with higher blood volumes and perfusion in patients with sickle cell anemia. (B–D) Example data from a single voxel for each component, whereby patterns evident in simulation are exhibited; representative group‐averaged slices at different relevant inversion times are shown as inserts. (E) Example kinetic modeling for the arterial and tissue compartments is shown for slice 54 and 76 of the 2 mm Montreal Neurological Atlas, as well as the residual of the fit at slice 76 which contains substantial voxels within the dural sinus and are separately modeled in this work. (F) Example arterial, gray matter, and venous masks used for quantification (see Methods for mask selection details).

**FIGURE 3:**
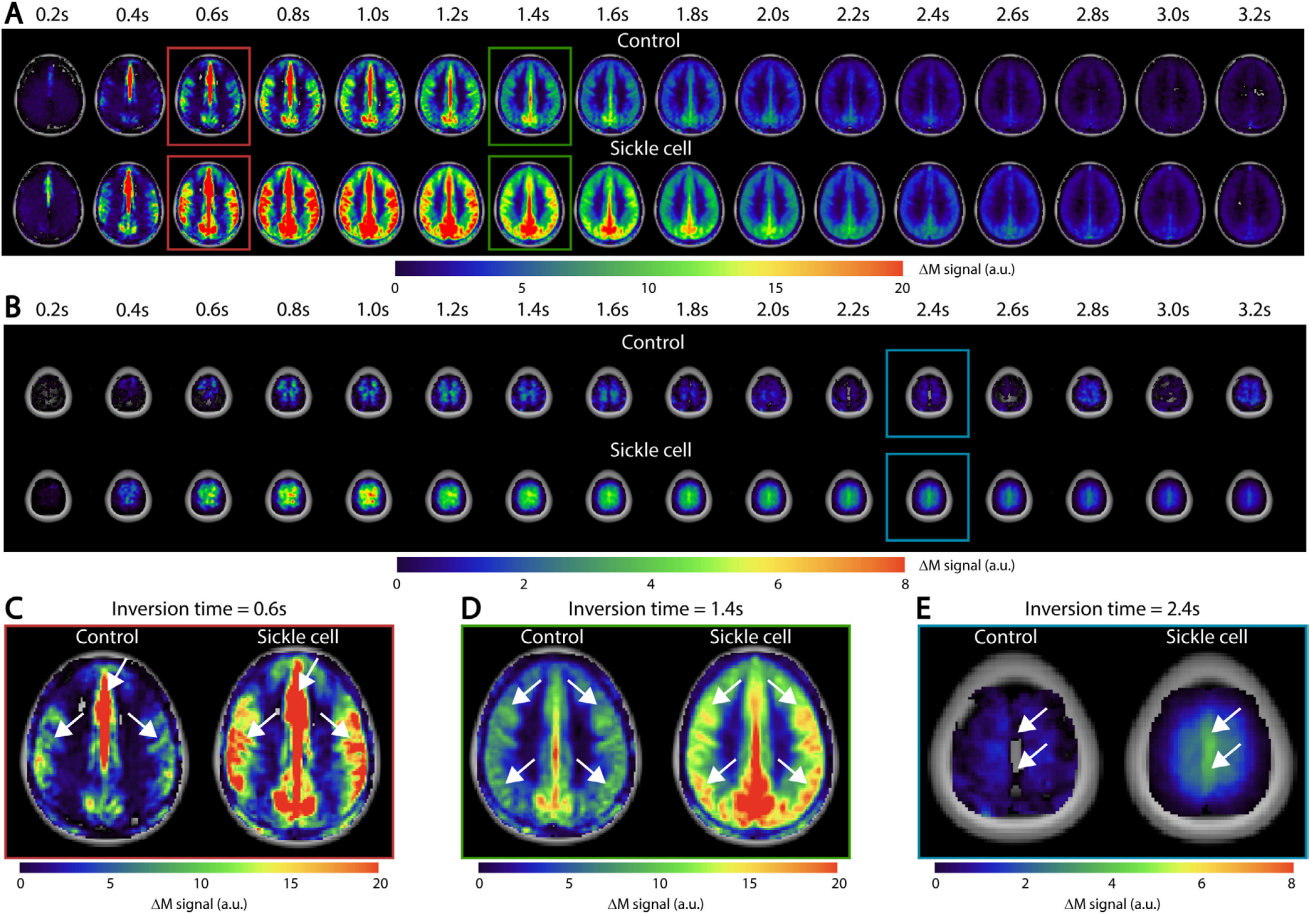
Group‐level blood inflow and perfusion maps for age‐ and sex‐matched nonanemic controls (*n* = 24) and patients with sickle cell anemia (*n* = 40). (A) One proximal slice (2 mm Montreal Neurological Institute, MNI, slice 54) and (B) one more distal slice (MNI slice 76) are shown to highlight regions with substantial arterial, gray matter, and dural sinus contributions. Magnifications of the early arterial flow signal indicated by white arrows (C), intermediate gray matter cerebral blood flow signal indicated by white arrows (D), and late sinus filling signal in sickle cell participants and absence of late sinus filling in control participants indicated by the white arrows (E) are shown below. Maps demonstrate evidence of earlier blood arrival times across components, as well as elevated blood volume and cerebral blood flow contributions in sickle cell anemia.

**FIGURE 4:**
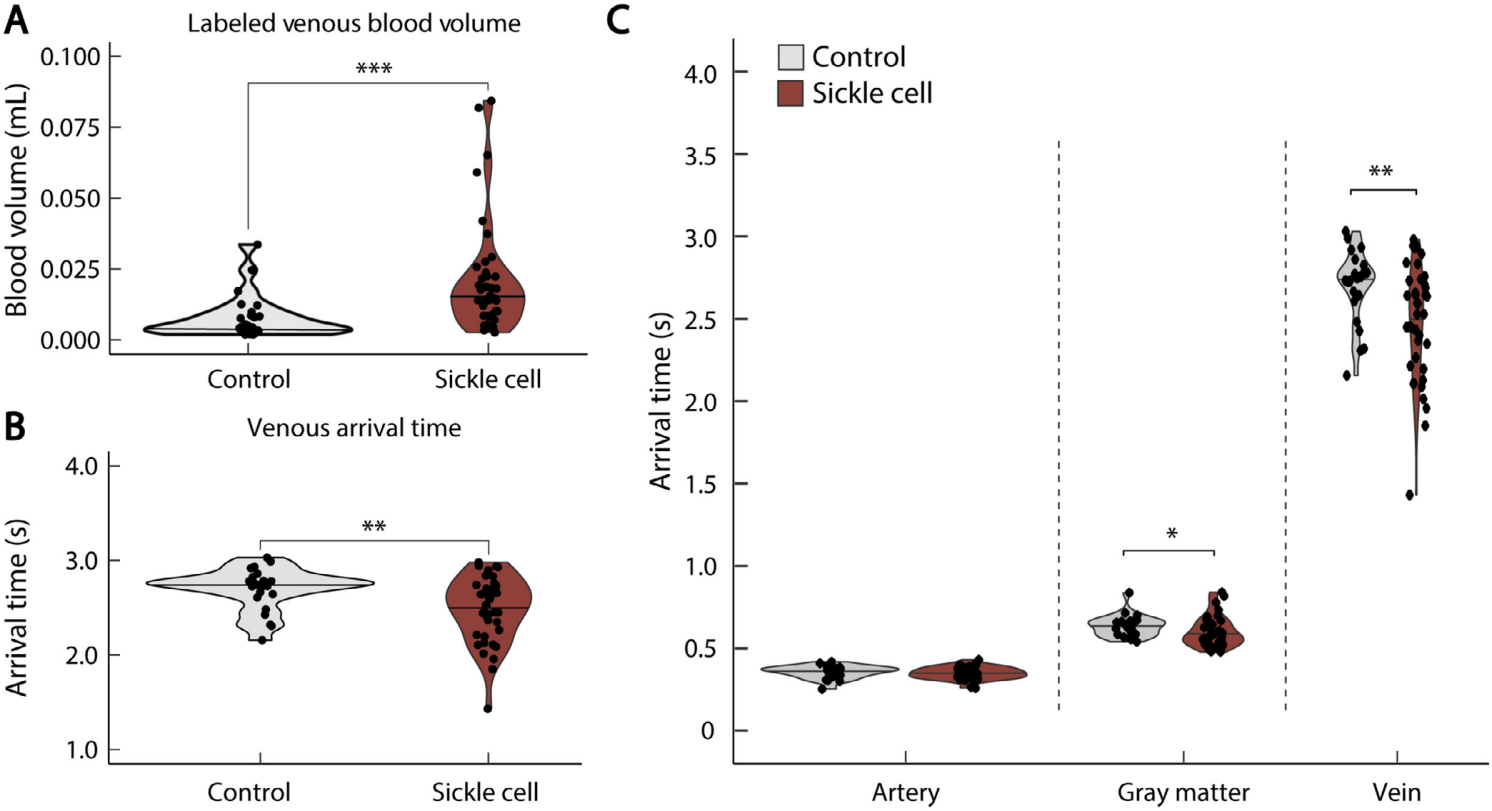
Quantification of venous hemodynamic parameters and transit times. Venous labeled blood volume is increased (***p < 0.001) (A) and venous arrival times reduced (**p < 0.01) (B) in sickle cell anemia relative to nonanemic control participants. (C) Arrival times across all components reveal that arterial arrival time is similar between groups and across participants, but gray matter arterial transit time (*p < 0.05) and venous arrival time (**p < 0.01) are both reduced in sickle cell relative to control participants. Data are plotted as violin plots with participant data points overlaid and horizontal lines denoting group median. Note that compared to arterial arrival time and gray matter arterial transit time, the distribution of venous arrival times is much larger across sickle cell anemia participants, which may motivate the relevance of this feature for the varied oxygen extraction and clinical trajectory in these patients.

Figure 1 shows image quality of the multi‐delay PASL sequence for a single participant; simulations demonstrating theoretical kinetic curves from Equations ((1), (2), (3)), group‐level measured kinetic curves, and regions‐of‐interest are shown in Figure 2. AAT was not significantly different (*p* = 0.371) between SCA (median = 0.35 s, IQR = 0.32–0.38 s) and control (median = 0.37 s, IQR = 0.33–0.38 s) participants. Gray matter ATT was shortened in SCA (median = 0.58 s, IQR = 0.54–0.65 s) compared to control (median = 0.64 s, IQR = 0.58–0.66 s) participants. VAT was decreased in SCA (median = 2.53 s, IQR = 2.23–2.72 s) compared to control (median = 2.75 s, IQR = 2.63–2.80 s) participants, whereas venous labeled blood volume was significantly increased in SCA (median = 0.015 mL, IQR = 0.0087–0.023 mL) compared to control (median = 0.0047 mL, IQR = 0.0032–0.0092 mL) participants. These findings were consistent with AAT being only marginally, nonsignificantly, reduced in patients with SCA, but tissue and VAT being significantly shortened. Group maps are shown in Figure 3 and quantitative values in Table 1 and Figure 4.

### 3.3 Transit Times and Ordinal Shunting Scoring

**FIGURE 5:**
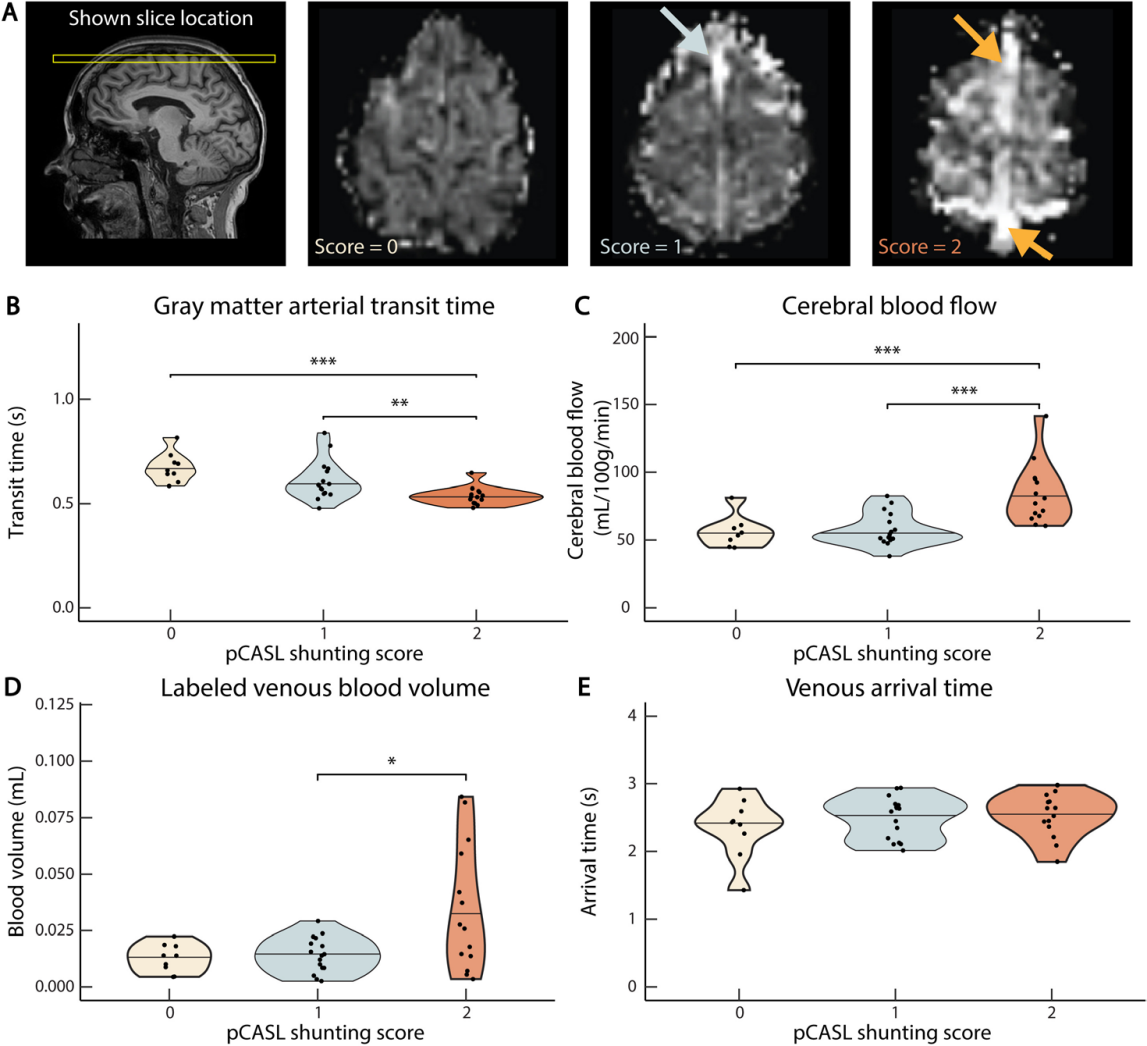
Continuous measures of vascular transit and cerebral blood flow versus ordinal shunting scores from single‐delay pseudo‐continuous arterial spin labeling (pCASL) data. (A) In the literature, so‐called venous shunting scores were made on single‐delay pCASL data, whereby score = 0 indicated no dural sinus hyperintensity, score = 1 indicated focal dural sinus hyperintensity near the crown, and score = 2 indicated diffuse dural sinus hyperintensity extending through the sagittal sinus to the torcula. When the same participants here are analyzed with this historical score from simultaneously acquired pCASL data, it is observed that the arterial transit times to the gray matter are reduced (B), cerebral blood flow increased (C), and labeled blood volume increased (D) in those with the highest shunting scores. No significant finding was observed with venous arrival time (E), indicating that these scores are most related to the volume of the labeled venous blood and capillary transit times. *p < 0.05, **p < 0.01, ***p < 0.001.

Next, the relationships between higher ordinal hyperintensity scores assessed with pCASL using identical procedures as published previously [18, 24] and multi‐delay PASL venous signal measures (VAT and labeled venous blood volume) were analyzed. As expected, pCASL and PASL gray matter CBF were significantly correlated (*r* = 0.51) across both groups. The mean pCASL hyperintensity score was significantly greater in SCA (mean = 1.13 ± 0.77) versus control (mean = 0.50 ± 0.51) participants, consistent with prior reports [18, 20, 24]. ANOVA analysis revealed that in participants with greater hyperintensity scores, pCASL CBF was also increased (*F* = 11.46) across the range of increasing hyperintensity scores (Figure 5). Venous labeled blood volume was also significantly increased in participants with greater hyperintensity score (*F* = 6.26) and the gray matter ATT was significantly shortened in participants with greater hyperintensity score (*F* = 10.10), but the VAT was not significantly related to the venous hyperintensity score (*F* = 0.72, *p* = 0.49). These findings are consistent with higher dural signal on single delay pCASL scans being generally associated with higher CBF, shorter arrival time of blood water to tissue, and higher volumes of nonexchanging blood water to veins in SCA.

### 3.4 Transit Times and OEF

OEF analysis yielded evidence of increased OEF in SCA patients when data were evaluated with the bovine and hemoglobin F models. The hemoglobin AA model found no difference in OEF between groups (*p* = 0.28) whereas the abbreviated hemoglobin S model found significantly decreased OEF in SCA patients (Supporting Information). These findings were consistent with established trends for these models [19, 47]. In participants with SCA, OEF was not significantly related to gray matter ATT (*p* = 0.24, *r* = −0.19), VAT (*p* = 0.48, *r* = −0.12) or labeled venous blood volume (*p* = 0.85, *r* = −0.03); statistics are for the hemoglobin F model as it is a full calibration model calculated over an approximately anemic range; however, similar findings were observed for all models (Supporting Information). These findings are consistent with vascular transit in SCA not being closely related to OEF on average.

### 3.5 Clinical Indicators of Disease Severity

**FIGURE 6:**
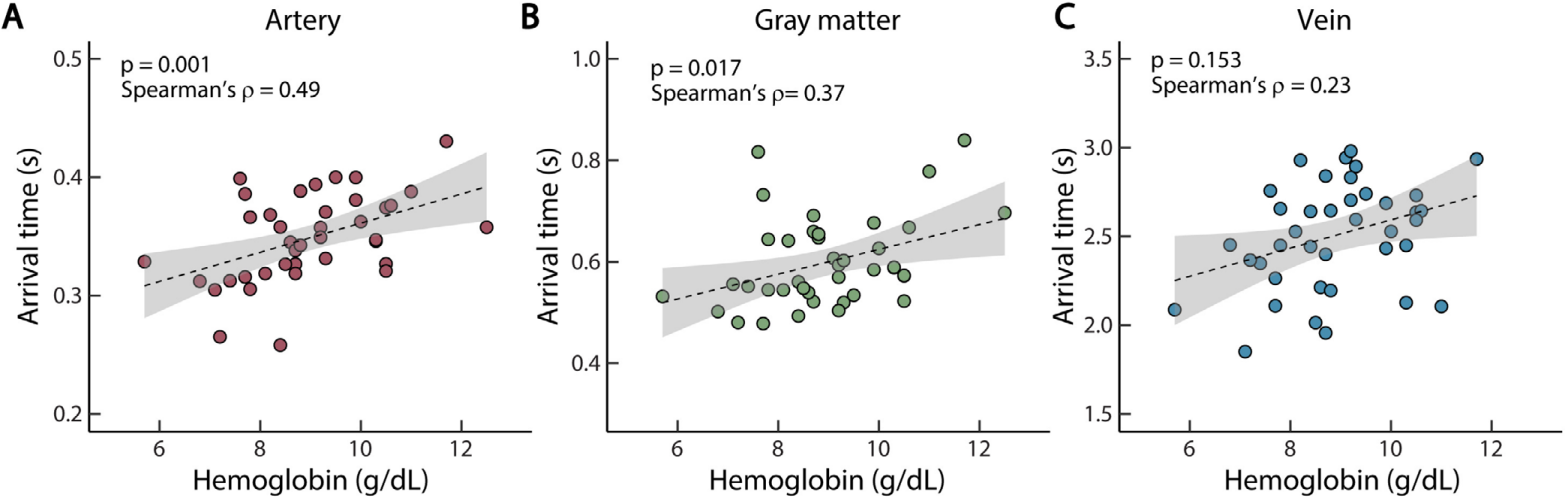
Relationships between arrival times and hemoglobin across different compartments. In sickle cell anemia participants, arterial arrival time (A) and gray matter arterial transit time (B) are both significantly related to total hemoglobin, but the venous arrival time is not significantly related to total hemoglobin (C). These findings are consistent with total hemoglobin influencing pre‐capillary blood kinetics, but additional regulatory mechanisms likely exist through the capillary bed to modulate transit and as such oxygen offloading, resulting in a weaker relation between venous arrival times and hemoglobin.

Finally, the relation between shortened vascular transit times and elevated shunted blood volume to clinical indicators of disease severity was assessed. Given the variable number of participants in each of the SCI groups, vascular transit and labeled venous blood volume was simply compared in participants with (*n* = 26) versus without (*n* = 14) prior infarcts. No vascular transit measure was significantly different between these participant groups. Next, transit measures were assessed for relation to total hemoglobin, a well‐known marker of condition severity (Figure 6). Here, AAT and ATT were positively associated with hemoglobin in SCA participants, yet the relationship with VAT was not significant (*p* = 0.153, *r* = 0.23). These findings are consistent with prior SCI history not being closely related to vascular transit in the enrolled cohort, and that the extent of anemia (i.e., total hemoglobin) relates most closely to arterial and tissue arrival times, but not to VATs.

## 4 Discussion

Multi‐compartment modeling of contrast from multi‐delay ASL MRI was performed to quantify blood transit across different vascular compartments in the setting of anemia. The major findings are that while AAT is similar in adults with versus without SCA, gray matter ATT and VAT were shorter in SCA relative control participants; additionally, quantitative labeled venous blood volume was increased in SCA relative to control participants and associated with venous hyperintensities on commonly‐acquired single delay ASL. The AAT and gray matter ATT were positively associated with total hemoglobin, yet no significant relationship was observed between VAT and hemoglobin. Findings are consistent with the hypotheses that SCA patients have decreased cerebral circulation times, increased imaging features consistent with aberrant blood‐tissue water exchange, and that regulatory mechanisms at the capillary level may reduce the dependency of vascular transit times on anemia extent to maintain adequate oxygen delivery to tissue, on average.

Findings are consistent with prior work from multiple groups that have observed evidence of increased dural sinus signal in patients with SCA [22, 23, 24]. Dural hyperintensities on ASL are fundamentally due to differences in blood‐tissue water exchange, which could be attributable to faster blood water transit or changes in blood–brain barrier permeability. Prior work has shown that these hyperintensities correlate with independent large‐vessel measures of flow velocity [24] and partially normalize following red cell exchange transfusion [19], highlighting relevance of flow velocities. In principle, blood–brain barrier permeability could also be affected, as has been proposed [22], although the multi‐delay nature of this work suggests that blood transit also is likely relevant.

More specifically, this study extended on these prior single‐delay ASL measures to quantify blood transit across different vascular compartments with multiple‐delay ASL. This study provides evidence in support of dural sinus hyperintensities correlating with reduced gray matter ATT, and that primarily gray matter ATT and VAT values are reduced in SCA relative to age‐ and sex‐matched nonanemic controls. Only one study has attempted to characterize the late venous signal in multi‐delay ASL in SCA patients [48]. Similarly to this study, the investigators found increased superior sagittal sinus signal in SCA patients relative to controls, but in contrast found longer superior sagittal sinus VAT in SCA patients relative to controls. This counterintuitive finding of longer VAT in the presence of increased CBF may be due to differences in modeling, as the prior study did not include separate terms for arterial and venous compartments.

Despite finding robust group‐level effects, there were no significant relationships detected with either OEF or prior infarct history. These findings help to inform the interpretation of dural sinus hyperintensities in the context of cerebral physiology. Capillary shunting in principle limits maximal OEF [15], hence, in regions and participants where oxygen delivery is not approaching a maximum ceiling, the existing OEF may be unrelated to increases in capillary shunting as measured by labeled venous blood volumes and VAT, as observed here. The measures made here were for near‐whole brain, and it is possible that shunting effects could be more prominently related to OEF in perfusion borderzone regions where infarcts are most common [11, 49]. Future studies could use regional OEF methods to quantify OEF and regional shunting phenomena to explore this possibility [50, 51]. Lastly, it is unknown when prior infarcts occurred. Infarcts may have occurred decades prior when treatments were not optimized and hemodynamic status differed from the time of measurement. In a recent prospective and longitudinal study of infarct development [52], preliminary evidence was provided to support dural sinus signal on single‐delay ASL as a marker of new infarct development, and as such, prospective studies to evaluate biomarker potential are needed.

This study was unable to reproduce previous results between hemoglobin AA model OEF and venous hyperintensity signal scoring [18]. This may be attributable to the smaller SCA cohort in this study (Juttukonda et al. [18], SCA *N* = 68; this study SCA *N* = 40) and patients from each study had different degrees of infarct history. Furthermore, the ASL readout from the previous study was 2D‐EPI [18] which would have allowed greater time for the labeled blood water to reach the superior sagittal sinus in more distal slices compared to the 3D‐GRASE readout utilized in this study.

Interestingly, hemoglobin was found to be positively associated with AAT and gray matter ATT, but weakly associated with VAT. Otherwise stated, the blood transit to large arteries and gray matter in SCA is colinear with the degree of anemia, and this relationship was weakened in the post‐capillary vasculature. This finding is likely consistent with the lack of an OEF relationship with venous hyperintensity score as summarized above, as it suggests regulatory capillary mechanisms to facilitate adequate oxygen extraction. Such an observation may be consistent with the active nature of blood flow regulation at the microvascular level, where pericytes or other active regulatory phenomena modulate red cell residency times in the capillaries to ensure adequate time for required oxygen delivery to tissue [15, 53, 54].

Several technical issues should also be considered. While venous bolus duration was a fit parameter given anticipated venous signal dispersion, for both tissue and arterial signal modeling, the bolus duration was fixed at 0.7 s and there was no bolus dispersion assumed. This determination was motivated by prior work using independent measures of cervical velocities from phase contrast angiography which observed only small differences in cervical flow velocities of approximately 15%, time‐averaged over the cardiac cycle, in SCA versus control participants [9]. Simulations indicated that this small change in arrival time would be difficult to accurately quantify given the temporal and spatial resolution of multi‐delay ASL. Future work could, however, test the effect of the cervical velocities in SCA patients on arterial flow dispersion and bolus length. In contrast, venous dispersion was readily detectable in patients and controls, generally manifesting as multiple discrete arrivals in some participants. As a result, the venous model included the option to fit multiple arrival peaks to the same voxel to incorporate this finding. When the standard deviation of these fits was considered, a trend (*p* = 0.075) was observed for the standard deviation increasing in SCA versus controls. As such, future work that investigates venous signal dispersion rigorously with more sophisticated models is likely warranted.

Given that SCIs are primarily localized to white matter parenchyma [49], white matter ATT may be additionally relevant. White matter perfusion signal has been shown to be reliable in SCA participants on the group‐wise level, but it is below the noise floor for the majority of inversion times recorded in this study [55]. This well‐characterized phenomena arises from both the fact that white matter CBF is 2–3 fold lower than gray matter CBF and often near the noise floor in ASL scans, and additionally, due to the white matter arrival times often being longer than the blood water *T*_1_ at 3T [55]. Given these limitations, this work focused on gray matter CBF and ATT where signal was most reliable.

### 4.1 Limitations

First, PASL with a 2D EPI Look‐Locker readout was used to quantify venous shunting, rather than pCASL, as the shorter labeling duration of several milliseconds in a pulsed method increases sensitivity to vascular transit effects compared to what can be detected with the long 1.5–2 s flow‐driven labeling pulse train in pCASL. SNR with PASL is reduced relative to pCASL, and as such more signal averages are acquired to increase SNR. Second, this is a single center study, whereas a multi‐center study with a larger cohort may provide more generalizable results. Third, volume coverage was confined to extend from the most distal aspect of the superior sagittal sinus through the bi‐parietal and bi‐frontal lobes. This decision was based on the signal of interest, venous intravascular signal, and population of interest, SCA, where infarcts are well‐characterized to occur in frontal and parietal lobes and anterior flow territories. Gray matter ATT values are within range, albeit on the shorter range, of whole‐brain literature values, as some posterior flow territory parenchyma where gray matter ATT is lengthened was not assessed. Finally, prior infarct presence was characterized in 40 SCA patients cross‐sectionally, but future work is needed to follow patients longitudinally to evaluate how these findings may portend future infarct development.

## 5 Conclusions

This study applied multi‐delay ASL MRI over a wide range of possible vascular transit times and provides evidence for reduced venous blood arrival time and increased labeled venous blood volume in SCA patients. Findings also indicate that the extent of anemia is closely related to arterial and tissue blood arrival times, but less significantly for post‐capillary transit times, suggesting that additional capillary regulatory mechanisms are present in many SCA patients to preserve oxygen extraction even in the setting of hyperemia. Future work is required to understand this regulatory mechanism and whether dysfunction contributes to future infarcts in this population.

## Funding

This work was supported by the National Heart, Lung, and Blood Institute (R01HL155207), National Institute of Neurological Disorders and Stroke (R01NS123281), National Institute on Aging (F32AG097081).

## Disclosure

The authors have nothing to report.

## Supporting information


**Table S1:** Oxygen extraction fraction (OEF) values.
**Table S2:** Oxygen extraction fraction (OEF) correlations with shunting and bolus arrival time (BAT) measures.
**Table S3:** Sickle cell anemia patients without moyamoya.
**Table S4:** Sickle cell anemia patients with moyamoya. Note that the *p*‐values should be interpreted cautiously, given the very small sample size of the sickle cell patients with moyamoya (*n* = 5). The values are primarily intended to show how the median BAT measures vary between patients with moyamoya, controls, and patients without moyamoya (e.g., Table S3).

## Data Availability

Full imaging parameter files and source data are made available from our laboratory Web site: biosightllc.com/academic.
