# Supplementary material for: Cerebral Blood Transit in Sickle Cell Anemia
Source: J Magn Reson Imaging. 2026 May 28;64(3):738–50. doi: 10.1002/jmri.70382 (PMC13387424; doi:10.1002/jmri.70382)
Supplement: Supplementary file 1 [file JMRI-64-738-s001.docx]

**SUPPLEMENTAL INFORMATION FOR:**

**CEREBRAL BLOOD TRANSIT IN SICKLE CELL ANEMIA**

**SUPPLEMENTAL METHODS**

The below sections provide additional information on the details of image registration, partial volume correction, mask selection, and model fitting.

*Image registration*

For purposes of registration between images with different contrasts, *T*­_1_-weighted images were bias corrected, brain extracted, and tissue segmented using advanced normalization tools (ANTs)^1^ and the FMRIB Software Library (FSL)^2^. Skull stripping was performed using FSL-BET^3^ and partial volume estimation images from FSL-FAST were used to create separate total white and gray matter masks with a threshold of 0.8 for each tissue type^4^. *M*_0_ images from the associated PASL and pCASL acquisitions were skull-stripped and linearly registered to the native *T*_1_-weighted image of the participant, and the *T*_1_-weighted images were subsequently non-linearly registered to an MNI152 *T*_1_-weighted 2 mm atlas^5^ with ANTs^1^. pCASL *M*_0_ and PASL *M*_0_ to standard space transforms were obtained by concatenating the transformation matrices and these matrices were used to transform the arterial spin labeling (ASL) data to standard space. Of note, all fitting was performed in the native space of the ASL images, and the above registration procedures were simply to allow visualization of identical structures across participants, as well as for group averaging and spatial visualization of group patterns.

*Mask selection for vascular compartments*

Gray matter bolus arrival time (BAT) values pertain to those calculated from a gray matter mask, whereas the intravascular (i.e., arterial and venous) BAT values were calculated in regions with high blood volume fraction for each compartment, as outlined by the following procedures.

The gray matter mask was chosen according to the criteria outlined in the prior section, using standard outputs from the FSL-FAST segmentation with a tissue type segmentation of 0.8. An example of this mask is included in Figure 2 of the manuscript.

The intravascular fitting was performed voxel-wise in the native space of the participant, and the arterial BAT was calculated within voxels in which the arterial blood volume fit was more than three standard deviations above zero; this procedure was performed in an effort to increase specificity for voxels with predominately arterial intravascular signal and reduce partial volume effects from parenchyma with small arterial blood volume fractions (e.g., typical parenchymal blood volume is 5% of voxel, with arterial blood volume representing 20-30% of this total blood volume). Mean, median, and interquartile ranges for values in these arterial masks were reported in the study; an example mask is shown in Figure 2 of the manuscript. As such, the arterial BAT values reported in this work can be interpreted as the BAT of voxels that contain predominately feeding arteries, rather than the initial arterial arrival within parenchymal voxels that contain only a small amount of arterial blood volume.

The venous masks were also based on criteria for voxels comprising a significant proportion of blood, albeit venous blood in this case. In preliminary work, we investigated multiple approaches for voxel selection by comparing each with dural sinus localization. The approach that was selected as most robust considers the modeled time series of the difference magnetization (ΔM) from FSL-BASIL, which incorporates arterial and tissue compartment modeling. It was identified that the residual signal from this fit is well-localized to the dural sinus on independent angiography and *T*_1_-weighted approaches (e.g., see Figures 1-2 of the manuscript text). As such, the mean measured ΔM signal was subtracted from the modeled ΔM signal from BASIL to estimate the residual ΔM, which provided an estimate of the venous signal localization. An example of this map is provided in Figure 2 of the manuscript. The group residual ΔM signal was transformed into standard space and used to identify a continuous superior sagittal sinus mask based on the signal intensity of the SCA groupwise mean residual signal in the standard space (Figure 2 within the manuscript). The same, identical venous mask was used in all participants to reduce variability from individual participant voxel selection. Importantly, the venous mask is localized to the dural superior sagittal sinus, and we did not attempt to quantify transit times in smaller, draining veins which often have signal at or below the noise floor.

As such, the BAT values in this work can be interpreted as arising from (i) voxels that contain primarily larger arterial blood volume and calculated on an individual participant basis in the case of arterial BAT, (ii) the total gray matter parenchyma for gray matter BAT which were also calculated on an individual participant basis, and finally (iii) a conserved, common region within the superior sagittal sinus for assessing venous BAT.

*Cerebral blood flow and venous hyperintensity scoring*

The goal of this component of the study was simply to compare the novel, multi-delay ASL measures of BAT with simpler procedures that have been reported in the literature using single-delay pCASL. As such, we complemented the multi-delay ASL scan with a single-delay pCASL 3D-GRASE scan and applied identical scoring procedures to the pCASL 3D-GRASE as reported in the literature in other studies.

Specifically, cerebral blood flow from the pCASL 3D-GRASE data was quantified using a two-compartment model^6^ with blood *T*_1_ estimated using the hematocrit from venipuncture and the calibration model estimated by Lu et al. 2004^7^ and mean cohort labeling efficiencies and BAT values determined by Juttukonda et al. 2019 (α_control_=91%, α_SCA_=72%; BAT_control_=1.13s, BAT_SCA_=1.01s)^8^. The venous hyperintensity score was calculated by applying a scoring system from the literature to the pCASL CBF image^9^, whereby score=0 is no apparent hyperintense signal in the superior sagittal or straight sinus, score=1 is focal signal near the distal aspects of the superior sagittal sinus that does not extend to the torcula, and score=2 is sagittal or straight sinus hyperintensity extending proximally to the level of the torcula^9^. Examples of each score are shown in Figure 5 within the manuscript text.

*Venous fitting*

We fit the venous signal using the intravascular blood relaxation model in Eq. 3 from the main text of the *Methods* section. We constrained the fits with the following parameters: venous BAT: 1.4-3.2s and venous bolus length (t): 0.2s-0.8s using the *lsqcurvefit* function in MATLAB. After reviewing the data and fitting procedure, we concluded that to capture the venous arrival to the superior sagittal sinus, we required the flexibility to fit multiple boluses in a given venous voxel in some participants. To do this, we first fit the model with a single intravascular bolus, then we fit a model including two separate intravascular arrivals with separate possible venous BAT values, venous bolus lengths, and venous shunted blood volumes. To determine the appropriate model, single or double arrival, the goodness of fit was estimated from an R^2^ for each model. If the R^2^ for the double arrival model explained at least 5% more of the variance than the single arrival model, the double arrival model was selected, otherwise the single arrival model was selected. To estimate the venous shunted blood volume from each participant, the significantly fit voxels were summed for each arrival and voxel. Finally, to estimate the venous BAT for each participant, the mean venous BAT across every significantly fit voxel and arrival was weighted by that arrival’s venous shunted blood volume. This was performed to account for the venous BAT to the superior sagittal sinus considering the relative volume of each bolus of labeled blood water.

**SUPPLEMENTAL RESULTS**

*Oxygen extraction fraction results*

OEF values are reproduced below, using different models for conversion of the measured venous *T*_2_ to venous oxygenation and subsequently OEF:

|  | Sickle cell | Control | p-value |
| --- | --- | --- | --- |
| **Demographic characteristics** | | | |
| Number | 40 | 24 |  |
| Sex (% male) | 47.5 | 37.5 | p=0.440 |
| Age (years) | 26.5 (22.5-32.0) | 29.5 (25.5-34.5) | p=0.125 |
| **Oxygen extraction fraction** | | | |
| Full bovine model (%) | 44.0 (41.1-48.3) | 34.7 (32.6-38.7) | p<0.001 |
| Abbreviated HbA model (%) | 40.2 (37.9-43.1) | 38.0 (36.4-41.8) | p=0.278 |
| Full HbF model (%) | 44.0 (42.0-47.4) | 37.9 (36.3-41.8) | p<0.001 |
| Abbreviated HbS model (%) | 29.7 (25.9-31.9) | 38.0 (36.4-41.8) | p<0.001 |

**Supplemental Table 1: Oxygen extraction fraction (OEF) values.**

OEF was significantly different in sickle cell anemia (SCA) participants using all models except the hemoglobin-AA model. Importantly, while all non-anemic controls had HbAA and patients had a mix of HbS, HbA, and HbF depending on treatment, the mathematical models themselves have different terms, and as such the difference in possible accuracy of all models is not simply a result of the hemoglobin type, but also the hemoglobin type and the terms included in the published models. For instance, both the bovine and HbF models were calculated using a full CPMG decay model (including three model coefficients), albeit over a normal or slightly anemic hemoglobin range. By contrast, the HbS and HbAA models were calculated over an anemic hemoglobin range, but did not include all model terms. It is well-known that the HbS model provides OEF values that are lower than all other models, prior PET literature, and physiological expectations; however, within the field there is no consensus on which model is most accurately currently. As such, we provide all values here for completeness and it should be noted that the study findings which pertain primarily to BAT and the relationship between BAT and clinical characteristics are not dependent on the above assumptions.

Finally, we also performed pairwise Spearman rank assessments between the four different models whereby OEF was the dependent variable and BAT or venous shunted blood volume in sickle cell anemia patients (N=40) were independent variables. Spearman’s r values are shown below with two-sided p-values shown below.

|  | *HbAA Model OEF* | *HbF Model OEF* | *Bovine Model OEF* | *HbS Model OEF* |
| --- | --- | --- | --- | --- |
| Gray matter BAT (s) | r=-0.11  p=0.49 | r =-0.19  p=0.24 | r =-0.24  p=0.13 | r =0.09  p=0.56 |
| Venous BAT (s) | r =-0.07  p=0.68 | r =-0.12  p=0.48 | r =-0.12  p=0.46 | r =-0.01  p=0.97 |
| Venous shunted blood volume (mL) | r =-0.10  p=0.55 | r =-0.03  p=0.85 | r =0.02  p=0.89 | r =-0.13  p=0.44 |

**Supplemental Table 2: Oxygen extraction fraction (OEF) correlations with shunting and bolus arrival time (BAT) measures.**

*Bolus arrival time in the setting of moyamoya vasculopathy*

We included patients meeting inclusion criteria with SCA and varying levels of clinical impairment. A subgroup of these patients had flow-limiting moyamoya syndrome, a not uncommon comorbidity in patients with SCA. Importantly, moyamoya comprises the non-atherosclerotic steno-occlusion of intracranial segments of primarily the anterior circulation, including the intracranial internal carotid arteries, as well as the middle and anterior cerebral arteries and their distal branches. The corresponding development of lenticulostriate or leptomeningeal collaterals, hallmarks of moyamoya, may stand to lengthen blood arrival in a manner directionally opposite to anemia.

In our cohort, 5 patients had moyamoya. The gray matter BAT for these patients was median=0.66s (IQR=[0.63-0.75s]) compared to median=0.57s (IQR=[0.53-0.64s]) for patients without comorbid moyamoya. Below we report values in patients with vs. without comorbid moyamoya, as well as how significance between non-anemic controls and patients varies:

|  | Sickle cell  (non-moyamoya) | Control | p-value |
| --- | --- | --- | --- |
| **Demographic characteristics** | | | |
| Number | 35 | 24 |  |
| Sex (% male) | 48.6 | 37.5 | p=0.400 |
| Age (years) | 26.0 (21.3-33.5) | 29.5 (25.5-34.5) | p=0.144 |
| **Bolus arrival time (BAT)** | | | |
| Arterial BAT (s) | 0.35 (0.32-0.37) | 0.37 (0.33-0.38) | p=0.327 |
| Gray matter BAT (s) | 0.57 (0.53-0.64) | 0.64 (0.58-0.66) | p=0.005 |
| Venous BAT (s) | 2.59 (2.38-2.72) | 2.75 (2.63-2.80) | p=0.017 |
| Shunted venous blood volume (mL) | 0.0178 (0.0106-0.0234) | 0.0047 (0.0032-0.0092) | p<0.001 |

**Supplemental Table 3. Sickle cell anemia patients without moyamoya.**

|  | Sickle cell  (moyamoya) | Control | p-value |  |  |
| --- | --- | --- | --- | --- | --- |
| **Demographic characteristics** | | | |  |  |
| Number | 5 | 24 |  |  |  |
| Sex (% male) | 40.0 | 37.5 | p=0.917 |  |  |
| Age (years) | 27.0 (25.8-28.5) | 29.5 (25.5-34.5) | p=0.369 |  |  |
| **Bolus arrival time (BAT)** | | | |  | **Bolus arrival time** |
| Arterial BAT (s) | 0.34 (0.33-0.39) | 0.37 (0.33-0.38) | p=0.977 |  |  |
| Gray matter BAT (s) | 0.66 (0.63-0.75) | 0.64 (0.58-0.66) | p=0.371 |  |  |
| Venous BAT (s) | 2.20 (1.93-2.39) | 2.75 (2.63-2.80) | p=0.010 |  |  |
| Shunted venous blood volume (mL) | 0.0088 (0.0042-0.029) | 0.0047 (0.0032-0.0092) | p=0.285 |  |  |

**Supplemental Table 4. Sickle cell anemia patients with moyamoya.** Note that the p-values should be interpreted cautiously, given the very small sample size of the sickle cell patients with moyamoya (n=5). The values are primarily intended to show how the median BAT measures vary between patients with moyamoya, controls, and patients without moyamoya (e.g., Supplemental Table 3).

**SUPPLEMENTAL DISCUSSION**

*Clinical relevance and future directions*

While we observed a relationship between the bolus arrival time (BAT) metrics and the severity of anemia (e.g., Figure 6 of manuscript), we did not observe significant relationships between prior infarcts and the vascular transit measures. This is not entirely unexpected, as the development of infarcts may have occurred at any time in life, including early in life prior to the initiation of hydroxyurea or transfusion therapy. As such, the hemodynamic status at the time of imaging, relative to when the infarct developed, may be entirely different. Future work that evaluates how these measures relate to future infarct development are ongoing as part of a prospective, longitudinal clinical study at our center.

However, it is useful to consider the possible interpretations between the BAT measures and extent of anemia across vascular compartments (e.g., Figure 6 of the manuscript). Specifically, these findings suggest a significant relationship between the extent of anemia and BAT with the arterial and tissue transit times, but less so with the venous transit times. One explanation for this occurrence could be that the venous transit times are more diffuse, with multiple veins contributing to arrival peaks in the assessed superior sagittal sinus region. Furthermore, cerebral venous anatomy itself is known to be much more variable across individuals^10^. However, an additional explanation could relate to the ability of the capillaries themselves to regulate flow in the setting of anemia to maintain approximately normal oxygen extraction fraction, as observed here.

Specifically, pericytes are known to play a crucial regulatory role for adjusting capillary blood flow, which occurs through complex contractions along junctions within the capillary bed^11^. Such cells are in close proximity to capillary endothelial cells and actively constrict or dilate capillary diameter in response to stimuli and oxygen requirements^11^. Pericytes are known to respond to neurotransmitters, vasoactive substances, local metabolic signals, and changes in oxygen tension. It is logical that such cells help to modulate and distribute flow in the setting of hyperemia to ensure adequate time for oxygen offloading^12^. Dysfunction of pericyte-mediated flow regulation has been implicated in a range of neurological diseases, and it is possible that such dysregulation may develop in a subgroup of patients with anemia who lack abilities to regulate capillary flow fully, possibly as capillary endothelial function declines across the lifespan^12,13^. Ongoing work is focused on understanding whether the development of future ischemic damage is related to reduced venous arrival times.

Related to the above possibility, capillary transit time heterogeneity (CTH) has been reported to significantly impact tissue oxygen extraction^14–17^. When red blood cells traverse capillaries with highly variable transit times, the overall oxygen extraction becomes less efficient compared to a more uniform flow distribution. This is known to occur because the relationship between oxygen extraction and blood transit time is nonlinear: capillaries with very short transit times do not allow sufficient time for oxygen to diffuse into tissue, while exceedingly long transit times deliver oxygen until the hemoglobin is desaturated. The result is that increased CTH reduces the maximal oxygen extraction capacity. This phenomenon may have particular relevance in patients with SCA, where, as described above, capillary flow patterns are dynamically regulated by both arteriolar tone and pericyte-mediated capillary constriction. Pathological increases in CTH have been observed in conditions such as Alzheimer's disease and following ischemic injury, where pericyte dysfunction and capillary constriction increase CTH^15,18^. Functionally, this means that simply maintaining adequate bulk blood flow, or even high blood flow as in the case of SCA, may be insufficient to ensure proper tissue oxygenation if the spatial and temporal distribution of that flow becomes too heterogeneous, highlighting the importance of microvascular regulation beyond traditional measures of cerebral perfusion alone.

Interestingly, in the present work we observed that the oxygen extraction fraction was not closely related to the venous BAT, however, a large range of venous BAT values was observed across patients (e.g., Figure 4 in manuscript). This highlights that, like many other hemodynamic relationships in SCA, that the breakdown of flow homogeneity maintenance mechanisms in the setting of anemia may have relevance for portending different clinical trajectories, although ongoing work is necessary to confirm this possibility.

**SUPPLEMENTAL REFERENCES**

1.	Avants BB, Tustison NJ, Stauffer M, et al. The Insight ToolKit image registration framework. *Frontiers in Neuroinformatics*; 8. Epub ahead of print 2014. DOI: 10.3389/fninf.2014.00044.

2.	Jenkinson M, Beckmann CF, Behrens TEJ, et al. FSL. *NeuroImage* 2012; 62: 782–790.

3.	Smith SM. Fast robust automated brain extraction. *Hum Brain Mapp* 2002; 17: 143–155.

4.	Zhang Y, Brady M, Smith S. Segmentation of brain MR images through a hidden Markov random field model and the expectation-maximization algorithm. *IEEE Trans Med Imaging* 2001; 20: 45–57.

5.	Grabner G, Janke AL, Budge MM, et al. Symmetric atlasing and model based segmentation: an application to the hippocampus in older adults. *Med Image Comput Comput Assist Interv* 2006; 9: 58–66.

6.	Dai W, Shankaranarayanan A, Alsop DC. Volumetric Measurement of Perfusion and Arterial Transit Delay using Hadamard Encoded Continuous Arterial Spin Labeling. *Magnetic Resonance in Medicine* 2013; 69: 1014–1022.

7.	Lu H, Clingman C, Golay X, et al. Determining the longitudinal relaxation time (T1) of blood at 3.0 Tesla. *Magnetic Resonance in Medicine* 2004; 52: 679–682.

8.	Juttukonda MR, Jordan LC, Gindville MC, et al. Cerebral hemodynamics and pseudo-continuous arterial spin labeling considerations in adults with sickle cell anemia. *NMR in Biomedicine* 2017; 30: 1–9.

9.	Juttukonda MR, Donahue MJ, Davis LT, et al. Preliminary evidence for cerebral capillary shunting in adults with sickle cell anemia. *J Cereb Blood Flow Metab* 2019; 39: 1099–1110.

10.	Ota T. Functional Cerebral Venous Anatomy from the Viewpoint of Venous Collaterals Part I, Supratentorial Superficial and Deep Venous System. *Stroke: Vascular and Interventional Neurology* 2024; 4: e001050.

11.	Peppiatt CM, Howarth C, Mobbs P, et al. Bidirectional control of CNS capillary diameter by pericytes. *Nature* 2006; 443: 700–704.

12.	Hall CN, Reynell C, Gesslein B, et al. Capillary pericytes regulate cerebral blood flow in health and disease. *Nature* 2014; 508: 55–60.

13.	Jordan LC, Richerson W, Aumann M, et al. Cerebral Blood Flow in Children and Adults With Sickle Cell Anemia. *Neurology* 2025; 105: e214227.

14.	Jespersen SN, Østergaard L. The roles of cerebral blood flow, capillary transit time heterogeneity, and oxygen tension in brain oxygenation and metabolism. *J Cereb Blood Flow Metab* 2012; 32: 264–277.

15.	Østergaard L, Engedal TS, Moreton F, et al. Cerebral small vessel disease: Capillary pathways to stroke and cognitive decline. *J Cereb Blood Flow Metab* 2016; 36: 302–325.

16.	Østergaard L, Engedal TS, Aamand R, et al. Capillary Transit Time Heterogeneity and Flow-Metabolism Coupling after Traumatic Brain Injury. *J Cereb Blood Flow Metab* 2014; 34: 1585–1598.

17.	Vestergaard MB, Iversen HK, Simonsen SA, et al. Capillary transit time heterogeneity inhibits cerebral oxygen metabolism in patients with reduced cerebrovascular reserve capacity from steno-occlusive disease. *J Cereb Blood Flow Metab* 2023; 43: 460–475.

18.	Østergaard L, Aamand R, Gutiérrez-Jiménez E, et al. The capillary dysfunction hypothesis of Alzheimer’s disease. *Neurobiology of Aging* 2013; 34: 1018–1031.
